# Understanding Consumer Attitudes and Awareness After Admission to a Toxicology Unit

**DOI:** 10.1111/1742-6723.70218

**Published:** 2026-01-22

**Authors:** Ingrid Berling, Patrice Albert‐Thenet, Hayley Anderson, Geoffrey K. Isbister

**Affiliations:** ^1^ Clinical Toxicology Research Group University of Newcastle Newcastle Australia; ^2^ Department of Clinical Toxicology Calvary Mater Newcastle Newcastle Australia

## Abstract

**Objective:**

Involvement of consumers is critical in the management of deliberate self‐harm. We aimed to review the consumer experience in clinical toxicology patient management and research.

**Methods:**

An anonymous, consenting non‐consecutive 12‐question survey was administered to toxicology in‐patients between April 28th and October 14th 2025.

**Results:**

From 440 presentations, 43 (9.8%) patients participated in the survey. The patients had a positive response to communication (91%, *n* = 39), confidence with the treating team (95%, *n* = 41), respect (93%, *n* = 40), and access to care (88%, *n* = 38), but only 11 patients (26%) expressed an interest in participating in further toxicology research.

**Conclusion:**

There was a high level of satisfaction with the toxicology services, but almost three quarters of patients do not want to engage in further consumer feedback research.

## 1 Introduction

There is an increasing need for consumer involvement in research and development of health policy, which is being expected more by funding bodies for grant submissions [1, 2]. There is very limited information on the experiences of patients (also referred to as consumers) with deliberate self‐poisoning. Effective management of these patients necessitates an understanding of their perspectives, attitudes and experiences during their hospital journey. Consumer engagement is particularly crucial in clinical toxicology, where the nature of the care can profoundly impact patient recovery and future mental health outcomes. The aim of this study was to capture the voices of toxicology consumers treated in a hospital toxicology unit, focusing on five major dimensions of their experience.

## 2 Methods

We recruited non‐consecutive admitted patients aged 16 years and older with a deliberate self‐poisoning presenting to the Hunter Area Toxicology Service between April 28 and October 14, 2025. Patients with unintentional poisoning, envenoming, accidential poisoning, and patients with a history of violence or deemed inappropriate (e.g., active psychosis) were excluded. Following an explanation and the ability to opt out, an anonymous 12‐question survey was administered to the remaining patients designed to explore five key categories: communication, patient perceptions, respect during treatment, access to appropriate care and willingness to engage in future research initiatives.

## 3 Results

There were 43 participants from 440 eligible deliberate self‐poisoning patients (approximately 9.8% response rate), with results displayed in Table 1. Self‐reported gender was mainly female, in 30 patients (70%), with two reporting as gender neutral. Five patients were from the LGBTQI+ community, two identified as elderly, and another two as adolescent/youth. Remote/rural and vulnerable was reported once each.

**TABLE 1:** Questionnaire questions, responses and five key categories: Communication, patient perceptions, respect during treatment, access to appropriate care and willingness to engage in future research initiatives.

Category	Question	Yes response (total = 43)
Communication	Did the treating team talk to you in a way that was easy to understand?	39 (91%)
	Did you get sufficient information about your diagnosis/afflictions?	38 (88%)
	Were you involved in decisions regarding your treatment?	38 (88%)
Patient perceptions	Do you have confidence in the treating teams* professional skills?	41 (95%)
	Did you perceive the hospital's work as well organised?	39 (91%)
	Overall, was the help and treatment you received at the hospital satisfactory?	41 (95%)
	Overall, what benefit have you had from the care at the hospital?	Support, Felt cared for, reassured, I feel better, That I'm not alone, I'm in the safest space well supported, They treated me kindly and with no judgement
	Do you believe that you were in any way given incorrect treatment (according to your own judgement)?	2 (0.05%)
Respect during treatment	Did you feel respected during your hospital admission?	40 (93%)
Access to appropriate care	Did you have to wait before you were admitted for services at the hospital?	5 (12%)
Willingness to engage in future research initiatives	Would you be interested in being involved in further research on the topic of toxicology as a consumer advocate?	11 (26%)

* It definies the treating team. Treating team: Those who have had main responsibility for examinations and treatment. Most often these are physicians, but some receive care from nurses, psychologists or other health or social workers.

Thirty‐nine (91%) patients reported that the treating team communicated in a comprehensible manner, and 38 (88%) felt involved in their treatment decisions. Forty‐one (95%) expressed confidence in their treating team's skills, whereas only two reported not feeling that they had received correct treatment. Forty (93%) felt respected during their hospital admission and 38 (88%) felt they were able to access timely care. Only 11 (26%) expressed an interest in engaging in further research as a consumer.

## 4 Discussion

Incorporating consumer feedback is essential in designing responsive healthcare services that address the specific needs of diverse patient populations, particularly those that are vulnerable, such as youth, rural populations and the elderly. An essential aspect of the patient experience is their perception of respect within the healthcare system [3]. We found that 93% of participants indicated feeling respected during their admission, reinforcing the significance of respectful communication in patient‐centred care. Moreover, 88% reported receiving timely treatment, indicating that hospital processes can effectively accommodate urgent care demands, a critical factor in the management of toxicological emergencies.

It is important to note the low rate of participation in our survey (< 10%), which is likely confounded by self‐selection bias, as well as excluding dangerous and aggressive patients. Three‐quarters of consumers expressed a disinterest in participating in further consumer research related to their toxicology care. This, combined with our low recruitment rate into the study, shows a reluctance to participate in consumer research during self‐poisoning presentations. In reverse, the positive responses to the consumer experience may be biased by selection numbers. Possible barriers to patient engagement in self‐poisoning research activities could be attributed to various factors such as perceived burden, emotional distress, or concerns regarding data privacy. Understanding these barriers is vital for developing more inclusive and engaging research protocols that resonate well with patients' needs and preferences.

The literature suggests that greater involvement of patients in research can lead to more patient‐centred outcomes and foster a sense of ownership over one's healthcare journey [4]. However, our findings indicate the necessity for healthcare professionals to explore innovative methods that encourage participation and address the perceived barriers, thereby enhancing patient engagement in research and improving care quality.

Our results reveal both a reassuring level of professional trust among patients and highlight how critical it is for healthcare providers to foster a supportive atmosphere that respects patient dignity and enhances their self‐identity during treatment, particularly in the setting of deliberate self‐poisoning.

## 5 Conclusion

Our study found that overall patients had a positive response to their toxicology admission, but the majority were not interested in further consumer engagement with toxicology research. This lack of interest in research requires improved communication of research to this consumer group and associated interested parties, such as family, friends and support workers.

## Author Contributions

G.K.I. and I.B. designed the study. G.K.I., I.B., P.A.‐T. and H.A. collected data. G.K.I. and I.B. analysed the data. All authors contributed to content and discussion. GI is guarantor of the paper.

## Funding

This work was supported by University of Newcastle Australia (G2401519).

## Conflicts of Interest

The authors declare no conflicts of interest.

## Data Availability

The data that support the findings of this study are available from the corresponding author upon reasonable request.
